# Rhizosphere inoculation of *Nicotiana benthamiana* with *Trichoderma harzianum* TRA1-16 in controlled environment agriculture: Effects of varying light intensities on the mutualism-parasitism interaction

**DOI:** 10.3389/fpls.2022.989155

**Published:** 2022-10-20

**Authors:** Bo Tan, Yihan Li, Dongzhou Deng, Hongli Pan, Yue Zeng, Xiao Tan, Wenhua Zhuang, Zhuo Li

**Affiliations:** ^1^ State Key Laboratory of Hydraulics and Mountain River Engineering, College of Water Resource & Hydropower, Sichuan University, Chengdu, China; ^2^ Sichuan Development Guorun Water Investment Co. Ltd., Chengdu, China; ^3^ Sichuan Academy of Forestry, Chengdu, China; ^4^ Key Laboratory of Water Saving Agriculture in Hill Areas in Southern China of Sichuan Province, Crop Research Institute, Sichuan Academy of Agricultural Sciences, Chengdu, China

**Keywords:** artificial light supply, mutualism, *Nicotiana benthamiana*, parasitism, stress resistance, *Trichoderma* inoculation

## Abstract

*Trichoderma* spp., a genus of fast-growing and highly adaptable fungi that form symbiotic relationships with plant roots, rendering them ideal for practical use in controlled environment agriculture. Herein, this paper aims to understand how the *Nicotiana benthamiana* with inoculation of *Trichoderma harzianum* strain TRA1-16 responds to light intensity variation. Pot experiments were conducted under low and high light intensities (50 and 150 μmol·m^-2^·s^-1^, respectively) and microbial treatments. Plant growth, physio-biochemical attributes, activities of antioxidant enzymes, and phytohormones regulation were investigated. The results showed that for non-inoculated plants, the reduction in light intensity inhibited plant growth, nitrogen (N) and phosphorus (P) uptake, chlorophyll a/b, and carotenoid content. *Trichoderma* inoculation resulted in 1.17 to 1.51 times higher concentrations of available N and P in the soil than the non-inoculated group, with higher concentrations at high light intensity. Plant height, dry weight, nutrient uptake, and antioxidant activity were significantly increased after inoculation (p<0.05). However, the growth-promoting effect was less effective under low light conditions, with lower plant height and P content in plants. We suggested that when the light was attenuated, the mutualism of the *Trichoderma* turned into parasitism, slowing the growth of the host plant. The application of fungal inoculation techniques for plant growth promotion required coordination with appropriate light complementation. The mechanisms of coordination and interaction were proposed to be incorporated into the biological market theory.

## Introduction

With accelerated urbanization, modern agriculture puts forward new requirements for agricultural industrialization, such as localization of food production, biosecurity, pest and drought mitigation, and year-round crop production (Benke and Tomkins, 2017; Engler and Krarti, 2021; Tan et al., 2021). Controlled environment agriculture (CEA), which is currently manifested as sustainable intensification (Pretty, 2018), is seen as a strategy to address these challenges. In CEA systems, practitioners create a suitable plant growth environment and improve the quality and yield by regulating environmental factors such as temperature, nutrients, light, and CO_2_ concentration (Kalantari et al., 2018; Farhangi et al., 2020). Aiming to obtain sustainability of CEA systems requires a high level of coordination between the environment and organisms (both plants and microbes), and it is possible to develop microbial inoculants for agricultural biotechnology (Berg, 2009; Sammauria et al., 2020). Co-regulation of plant growth and metabolism can enable novel strategies for future sustainable food security and a new green revolution (Li et al., 2018).

As a plant growth-promoting fungi (PGPF), *Trichoderma* is the fungus gaining attention from agricultural practitioners (Bononi et al., 2020), and *Trichoderma* inoculation has become an effective method to improve yield in CEA systems (Wipf et al., 2019). Studies showed that *Trichoderma* could promote the growth of various crops in the field, such as tomato, maize, and rice (Mahato and Neupane, 2018; Khoshmanzar et al., 2020; Singh et al., 2020). Because of the vigorous survival and reproduction ability, *Trichoderma* can efficiently enhance plant vitality by forming symbionts with crops (Guzmán-Guzmán et al., 2019). When the hyphae of the *Trichoderma* sense signals from the plants, they grow toward the host plant’s root and eventually coil around it or attach to it by forming hook-like structures (Chet et al., 1981; Cortés et al., 1998). Fungal mycelia excrete compounds that promote plant root elongation, thereby improving water availability (Harman, 2000; Shoresh et al., 2010) and nutrient uptake (De Jaeger et al., 2011; Singh et al., 2019; Bononi et al., 2020). In addition, *Trichoderma* produces phytohormones that enhance plant tolerance to abiotic stresses, thereby stimulating plant growth and productivity (Zhao and Zhang, 2015; Illescas et al., 2021; Kredics et al., 2021).

In addition to soil-based cultivation systems, numerous benefits are provided by hydroponic systems, which, if properly managed, can provide optimal water and nutrients to the roots, contributing to improved plant growth and yield and resource use efficiency compared to soil (Paradiso et al., 2017). However, the high consumption of chemical fertilizers in nutrient solutions and the sensitivity of closed hydroponic systems to salinity are issues that need to be addressed. The application of *Trichoderma* in hydroponic systems may facilitate more efficient nutrient use, environmental control, and avoidance of soil-borne diseases, thus further improving crop productivity in hydroponics (Lee and Lee, 2015; Tarroum et al., 2021). Research showed that in a hydroponic environment, *Trichoderma* hyphae was observed to grow between plant cell walls at an early colonization stage of 10 h, while by 24 h, the root surface was extensively colonized (Moran-Diez et al., 2015). Furthermore, seeds treated with *Trichoderma* were found to have improved growth and physiological performance in a hydroponic saline environment, as evidenced by constantly faster and more uniform seed germination compared to untreated seeds (Yasmeen and Siddiqui, 2018). Practices in hydroponic systems showed that inoculation with *Trichoderma* provided higher fresh leaf yields, resulting in a 15%-18% reduction in fertilizer use (Moreira et al., 2022; Oliveira et al., 2022).

*Nicotiana benthamiana* (hereafter *N. benthamiana*) is a good choice of an infested host plant, which belongs to the Solanaceae family and is a heterotetraploid plant containing 19 chromosomes. It is a widely used experimental host plant in current phytopathological studies because it can be infected by various pathogens, including bacteria, oomycetes, fungi and so on (Goodin et al., 2008). Hence, *N. benthamiana* can be employed for colonization by *Trichoderma* to obtain symbionts that are environmentally sensitive and responsive (Harman, 2011). However, since the effects of *Trichoderma* inoculation varied under different conditions, some studies have even found that *Trichoderma* negatively affected plants (Kredics et al., 2021). Symbiotic growth is a multifactorial process in which light plays an important role.

Light radiation controls the central processes of plant growth and development for aboveground plant parts, including germination, leaf proliferation and expansion, and flower bud germination (Feng et al., 2019). Reductions in light intensity could induce inhibition of metabolic potential of photosynthesis and cell proliferation, render a reduction in the internal and stomatal conductance of the leaves, and reduce the diffusion of CO_2_ to the leaves, further damaging the leaf growth and enlargement (Wu et al., 2017; Yang et al., 2017; Wu et al., 2018). Furthermore, light radiation energy is obtained through long-distance signal communication between different organs to regulate rhizodeposition and thus influence microbial colonization and metabolism (Hughes et al., 1999). Thus, smaller and thinner leaves of the plants were observed under low light conditions compared to full sunlight conditions (Wu et al., 2017). Konvalinková et al. (2015) showed that as the light intensity decreased (from 241.5 μ mol·m^-2^·s^-1^ to 69 μ mol·m^-2^·s^-1^), the plant biomass was significantly reduced, and the benefits of mycorrhizal growth and P uptake were lost at 69 μ mol·m^-2^·s^-1^. Gehring (2003) found that compared with high light intensity (157 μ mol·m^-2^·s^-1^), low light intensity (54 μ mol·m^-2^·s^-1^) inhibited the growth of seedlings, reduced the root-to-shoot ratio, and increased specific leaf area. Tester et al. (1985) found that a reduction in light radiation inhibited the root uptake of P (and plant growth was impaired at 20 μ mol m^-2^ s^-l^). The P content of infected plants was significantly higher than uninfected ones.

The antioxidant system of the plant would respond to light stress. Reactive oxygen species (ROS), such as singlet oxygen associated with photooxidative damage, can cause severe damage to the photosystems and put the entire photosynthetic machinery into jeopardy (Triantaphylidès et al., 2008; Das and Roychoudhury, 2014). To protect against ROS damage, plants have developed efficient antioxidant machinery, known as enzymatic and non-enzymatic scavenging systems (Scandalios, 1993). Indole-3-acetic acid (IAA) and gibberellic acid (GA) are essential for plant growth and development (Hazzoumi et al., 2014), and they are responsible for inducing plant stress tolerance and improving plant growth by controlling the activity of antioxidant enzymes to reduce oxidative damage (Khalid and Aftab, 2020). Superoxide dismutase (SOD) and peroxidase (POD) are essential enzymatic antioxidants for promoting the scavenging of ROS (Zhang et al., 2020). Elevated activities of SOD and POD facilitate the conversion of toxic H_2_O_2_ to water and oxygen, thus protecting plants from the toxic effect of oxygen free radical (Blokhina et al., 2003). Shao et al. (2014) revealed that plants adapt to shade conditions by increasing chloroplasts, grana and grana lamellae numbers, and higher SOD and POD activities. The balance between SODs and the different H_2_O_2_ scavenging enzymes is crucial in determining cellular homeostasis (Mittler et al., 2004), and plays a protective role in the functional structure of cells and photosynthetic apparatus (Gama et al., 2009).

It is a highly intricate process in symbiosis growth, nutrient transport, and substance exchanges under varying light intensities. Our understanding of the response of plant-*Trichoderma* symbionts to artificial light management is hampered by the fact that there is only a minimal number of available studies on the regulation of the growth processes, especially from a holistic perspective of symbiotic growth from light supply to rhizosphere microenvironment (Konvalinková and Jansa, 2016). This study aimed (1) to characterize the effect of *Trichoderma* inoculation on the growth, physiological, and biochemical traits of *N. benthamiana* under different light intensities and (2) to further reveal the mechanism of transition of mutualistic *Trichoderma* to parasitism during energy conservation of artificial light supply.

## Material and methods

### Experimental preparation

This experiment involved *N. benthamiana* plant cultivation with/without *Trichoderma harzianum* inoculation in pots. The method of *Trichoderma* isolation and purification was followed by Xu et al. (2022), and this procedure was performed before plant cultivation. Briefly, the *Trichoderma* strain was isolated from soybean monoculture soil by serial dilution technique on a *Trichoderma*-selective medium (TSM). For purification, the single spore of the putative *Trichoderma* colonies was transferred on potato dextrose agar (PDA) plates and cultured at 28°C. The mycelia of fungal isolates were collected from 7-day-old isolates on PDA dishes and then ground in liquid nitrogen with a disposable pellet pestle. The total genomic DNA of all isolates was extracted for molecular identification using an SP Fungal DNA Kit (Aidlab Biotech, Chengdu, China) according to the manufacturer’s protocols. *Trichoderma* species were therefore identified by amplifying the sequences of ribosomal DNA internal transcribed spacer. Prior to planting, suspension of *Trichoderma* spores was made for inoculation. The plates covered with green mycelia were rinsed with sterile water, and the liquid was filtered through two layers of gauze to obtain spore suspension. The concentration of conidia in the suspension was determined under a microscope using a blood counting chamber, and the concentration of conidia suspension was calculated as 6×10^7^ CFU/mL. The prepared *Trichoderma* strain was stored at 4°C before use.

### Seed germination

The substrate soil for seed germination and plant growth was a mixture of peat substrate (Pindstrup Mosebrug A/S, Ryomgaard, Denmark) and vermiculite (3:1, v:v). Before cultivation, the pH of the substrate soil was measured to be 5.36 ± 0.20, the dry matter content was 55 to 75 g·L^-1^, and the N-P-K fertilizer was 0.650 kg·m^-3^. Due to the low nutrient content of the substrate soil, the macro elements required by the plants were provided through the Murashige and Skoog (MS) solution during cultivation, including CaCl_2_ 332.02 mg/L, KH_2_PO_4_ 170.00 mg/L, KNO_3_ 1900.00 mg/L, MgSO_4_ 180.54 mg/L, NH_4_NO_3_ 1650.00 mg/L.

The *N. benthamiana* seeds were sterilized in sodium hypochlorite for 3 min, washed five times in sterile water, and soaked in distilled water at 4°C for 48 h. Each pot (10×10×9 cm) was filled with 100 g of substrate soil. Then the pots were soaked till the soil was saturated with water. Five seeds were sown in each pot and budded in an incubator. After five days of germination, all plants were moved to the laboratory. The seedlings with similar growth conditions were retained (one seedling in each pot), and then 15 mL of MS solution was added to each pot.

### Growth conditions and experimental design

All plants were kept under a light intensity of 150 μmol·m^-2^·s^-1^ for 14 days, and then the plants were divided into two groups for growth experiments at different light intensities. Considering the essential photosynthetic intensity for plant growth while stimulating plant interactions against *Trichoderma* colonization, the value of L1 = 50 μmol·m^-2^·s^-1^ was used as suggested by Carvalho et al. (2008). Meanwhile, the light intensity L2 = 150 μmol·m^-2^·s^-1^ opted for normal growth of *N. benthamiana* as suggested by Kotakis et al. (2010). When the plants were observed to have two true leaves, half of the pots under L1 and L2 were inoculated with *Trichoderma* spore suspension (denoted as Tri). 120 mL of the suspension was injected into the rhizosphere with a pipette. For non-*Trichoderma* inoculated plants (denoted as NTri), the same 120 mL of the medium as the Tri group was injected, but without *Trichoderma* spores. Each combination had four replicates. All the plants were grown in controlled conditions, with a temperature of 25°C–26°C, 16/8 h (light/dark) photoperiod, and relative humidity of 70%–80%. The plants were watered daily with distilled water to approximately 80% of the soil water holding capacity.

### Analysis of soil and plant samples

Plant height was measured from the root collar until the tip of the inflorescence, and the stem diameter above the root collar was measured for each plant before harvest by a vernier caliper. The leaf area was determined by the coefficient methods (at k=0.75) (Koubouris et al., 2018). Then the plants were harvested after five weeks of the growth period. The rhizosphere soil of each treatment was sampled and air-dried in a shady place. The shoot dry weight (SDW), root dry weight (RDW), and total dry weight (TDW) were measured by electronic analytical balance (OHAUS, PR124ZH/E, China). Specific leaf area (SLA=leaf area/leaf mass) and root-shoot ratio (RSR=dry weight of root/dry weight of aboveground part) were calculated.

Soil available N and P were determined using the alkaline hydrolysis diffusion method and molybdenum-antimony anti-spectrophotometric method. For available N, 1.00 g of dried soil sample was treated in a diffusion dish with 10 mL of 1.8 mol/L NaOH solution. After diffusion, the sample was absorbed by 3 mL of boric acid and titrated with 0.01 mol/L hydrochloric acid solution, and the content of alkali-hydrolyzable N was calculated as Eq.1.


Eq. 1 $$W_{\text{N}}=\frac{(V-V_{0})\times c\times 0.014}{m}\times 10^{3}$$

where the *W*_N_ is the content of alkali-hydrolyzable N (mg/g), the *V* and *V*_0_ (mL) are the volumes of hydrochloric acid used in the titration sample, and blank group, respectively. The *c* (mol/L) was the concentration of the hydrochloric acid standard solution, which was 0.01. The *m* (g) is the weight of the soil sample, which is 1 in this experiment, and the 0.014 (g/mmol) is the millimolar mass of the N atom.

For soil available P measurement, 1.00 g of dried soil sample was added into a 200 mL plastic bottle. 50 mL of ammonium fluoride-hydrochloric acid was added as the extracting agent, shaken at a frequency of 180 ± 20 rpm using a reciprocating constant-temperature water-bath shaker (SHA-B, Yoke 140 Instrument, China), dried, and filtered through a P-free filter membrane. 10 mL of filtrate, 10 mL of deionized water, and two drops of dinitrophenol indicator were added to a 50 mL volumetric flask, titrated with boric acid until the solution appeared yellow, then added 5 mL of Mo-Sb Anti chromogenic agent. Finally, deionized water was added to the endpoint of 50 mL. After 30 minutes, absorbance (at 700nm) was measured by spectrophotometry. The standard curve was employed to determine the P content corresponding to the measured absorbance value.

Plant N was determined using the Kjeldahl method (Yuen and Pollard, 1953), while P was determined by Mo-Sb anti-absorption spectrophotometry (Bao, 2000). Soil pH was measured by IQ 150 pH Meter (IQ Scientific Instruments, America). Peroxidase (POD) and superoxide dismutase (SOD) were analyzed using Assay Kits (purchased from Nanjing Jiancheng Co., Ltd.). 0.5 g FW of plant tissue was accurately weighed, 0.1 mol/L phosphate buffer was added, and grounded in the ice bath for 5 min. The tissue homogenate was centrifuged at 3500 r/min for 10 minutes, and the supernatant was used to determine the POD and SOD activities. Indole-3-acetic acid (IAA) and gibberellic acid (GA) were determined by enzyme-linked immunosorbent assay (ELISA). 1.0 g FW of plant tissue was weighed accurately, phosphate-buffered saline (PBS) (1:9, w:v) was added to make tissue homogenate, centrifuged for 10 min at 2000 r/min, and then 50 μL of supernatant was taken for determination of the phytohormones. For chlorophyll a (Chl a), chlorophyll b (Chl b) and carotenoid, 0.3 g FW of leaves were weighed, 95% ethanol, quartz sand, and calcium carbonate powder were added for grinding. The tissue homogenate was filtered, and the chlorophyll was determined by spectrophotometry. The water content of the plant samples was determined simultaneously during the test by the drying and weighing method, and the FW concentrations were converted to DW concentrations after obtaining the data.

### Statistical analysis

Numerous interdependent factors affected the physiological and biochemical processes of *N. benthamiana* under different light conditions and *Trichoderma* inoculation. Therefore, principal component analysis (PCA) was employed to reduce the dimensionality of the possibly correlated variables while retaining most of the information. Seventeen variables, including plant height, stem diameter, leaf area, SLA, shoot dry weight, soil physical and chemical properties (pH, N, and P), plant chlorophyll contents (Chl a, Chl b, and carotenoid), N and P in the shoot, activities of related enzymes (SOD, POD) and the contents of the phytohormones (IAA and GA), were considered in the present study. Two-way analysis of variance (ANOVA) was used to examine the effects of variable light intensity incorporating *Trichoderma* inoculation and the interactions between the two factors. Individual treatment was statistically evaluated using Duncan’s multiple range test to identify whether they differed significantly (p=0.05). All statistical analysis was performed using IBM SPSS Statistics 19.

## Results

### Plant growth and physiological traits

**Figure 1:**
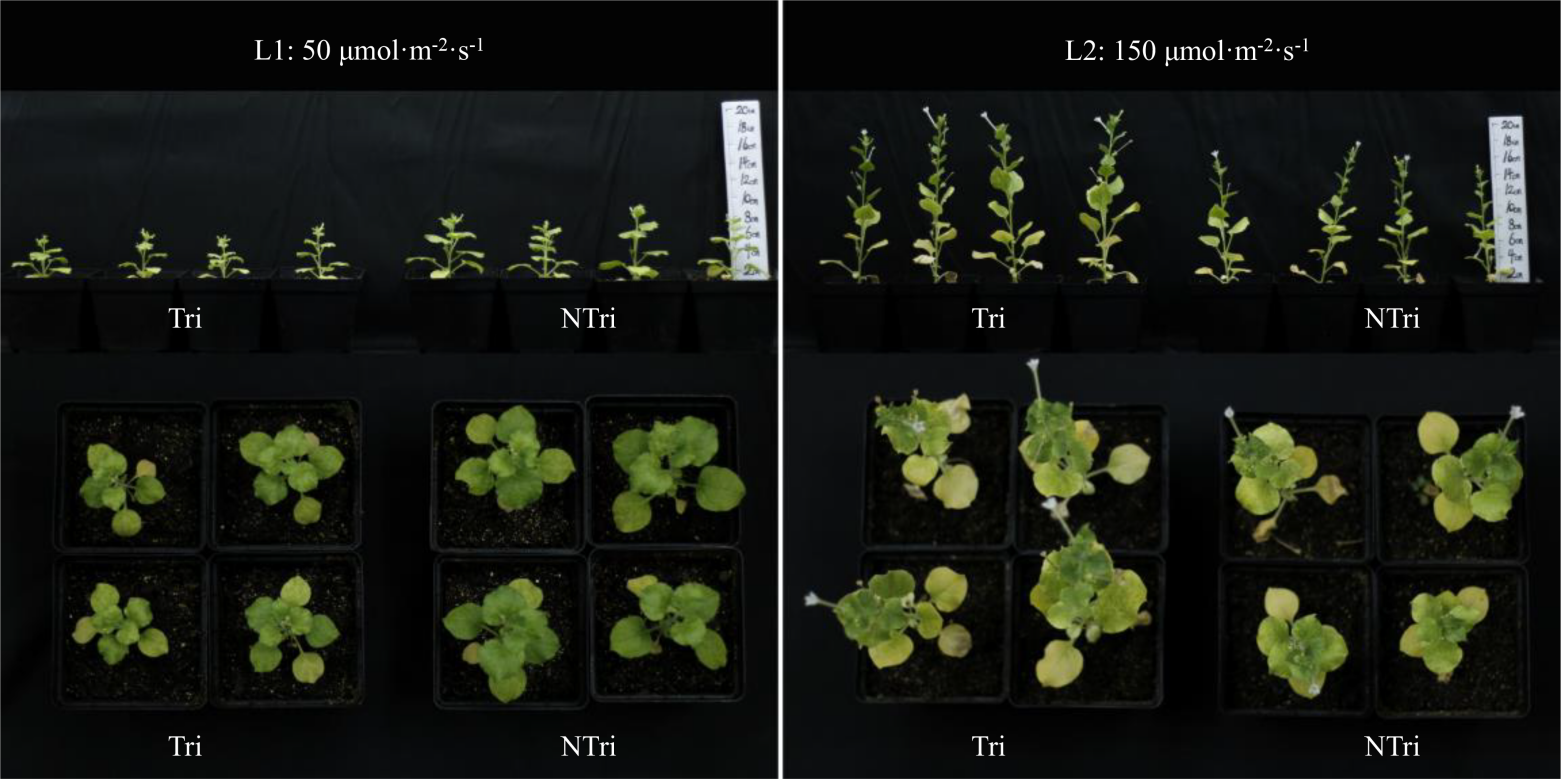
Comparison of the *N. benthamiana* growth under different light conditions with and without *Trichoderma* inoculation. L1 and L2 denote the two light intensities; Tri and NTri denote the treatment groups inoculated and not inoculated with *Trichoderma*, respectively.

**Figure 2:**
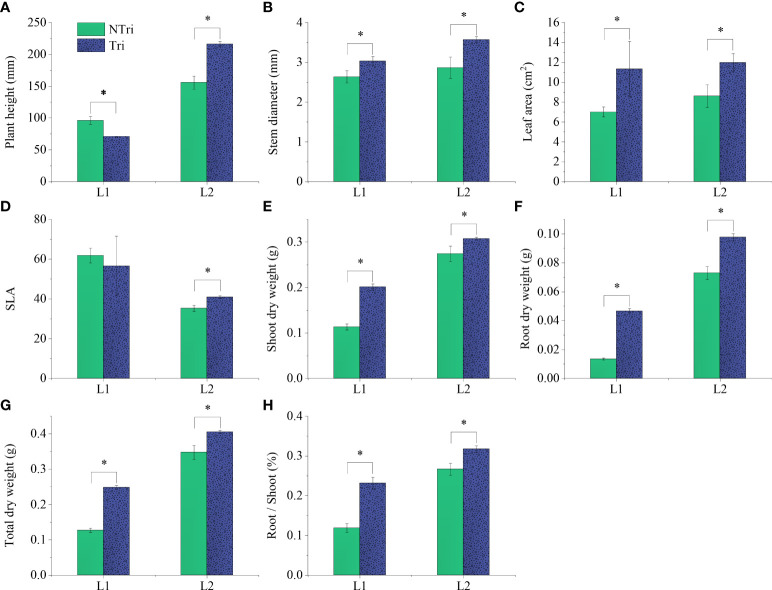
Comparing the plant height **(A)**, stem diameter **(B)**, leaf area **(C)**, specific leaf area **(D)**, shoot dry weight **(E)**, root dry weight **(F)**, total dry weight **(G)**, and root-shoot ratio **(H)** of the *N. benthamiana* inoculated or not with *Trichoderma*. L1 and L2 denote the two light intensities; Tri and NTri denote the treatment groups inoculated and not inoculated with *Trichoderma*, respectively. Error bars denote standard deviation, and asterisks indicate significant differences (p<0.05).

Significant differences in plant height could be visually observed. As shown in **Figure 1**, The plants grew slowly under L1 conditions due to insufficient light, and the plants were shorter in height in the Tri group compared to the NTri group. However, the results were inverted under L2 conditions, rendering the plants to grow normally, and the plants were taller in the Tri group compared to the NTri group. As shown in **Figure 2**, plant height in the Tri group (216.20 ± 4.20 mm) was 38.84% higher than in the NTri group (155.71 ± 10.23 mm) under the L2 condition. However, for the lower light intensity of L1, the plant height in Tri group (70.80 ± 0.62 mm) was 26.50% lower than in the NTri group (96.33 ± 6.11 mm). In the NTri group, the highest values of the stem diameter (2.86 cm) and leaf area (8.62 cm^2^) were found under the L2 condition; while in the Tri group, the stem diameter and leaf area were significantly increased by 24.64% and 38.81%, respectively compared with the NTri group. Under the L1 condition, the stem diameter and leaf area of the Tri group were significantly increased by 15.18% and 61.89% compared with the NTri group. The SLA of L1 was higher than L2 in both Tri and NTri groups. The SDW, RDW, RSR, and TDW for the Tri group under L1 were 1.78, 3.47, 1.95, and 1.95 times higher than the NTri group, respectively, and these parameters for the Tri group under the L2 were 1.12, 1.34, 1.19, and 1.17 times higher than NTri group, respectively.

As shown in **Table 1**, in the NTri group, the N content under the L2 condition (16.62 mg/g) was higher than that under L1 (13.11 mg/g). The N contents of the Tri group were 1.58 and 1.14 times higher than that of the NTri group under L1 and L2 conditions, respectively. The lowest P content (0.73 mg/g) was observed under the L1 condition in the NTri group. The P content for the Tri group under L2 was 1.65 times higher than the NTri group. Soil pH was significantly decreased by *Trichoderma* treatment both in L1 and L2 conditions. Meanwhile, the contents of soil available N and P for the Tri group under L1 were 1.17 and 1.28 times higher than the NTri group, while the contents of soil available N and P for the Tri group under L2 were 1.34 and 1.51 times higher than the NTri group.

**Table 1:** Physicochemical indices of soil and *N. benthamiana* under the treatment combinations of the two light intensities and *Trichoderma* inoculation.

Parameters	Treatment combinations*			
	L1 + NTri	L1 + Tri	L2 + NTri	L2 + Tri
Plant N (mg/g DW)	13.11 ± 0.03 d	20.74 ± 0.05 a	16.62 ± 0.02 c	18.91 ± 0.04 b
Plant P (mg/g DW)	0.73 ± 0.05 b	0.69 ± 0.02 b	0.90 ± 0.03 b	1.49 ± 0.02 a
Soil N (mg/g DW)	376.63 ± 22.56 a	420.42 ± 29.40 a	320.54 ± 19.20 b	408.19 ± 28.56 a
Soil P (mg/g DW)	56.29 ± 5.04 b	75.16 ± 6.00 a	45.73 ± 4.05 c	68.44 ± 5.44 a
Soil pH	5.67 ± 0.42 a	4.92 ± 0.39 c	5.59 ± 0.29 a	5.29 ± 0.25 b
Chlorophyll a (mg/g DW)	0.77 ± 0.02 b	1.03 ± 0.03 a	0.72 ± 0.02 b	0.74 ± 0.09 b
Chlorophyll b (mg/g DW)	0.39 ± 0.08 a	0.37 ± 0.04 a	0.23 ± 0.05 b	0.27 ± 0.03 b
Total chlorophyll content (mg/g DW)	1.17 ± 0.07 b	1.40 ± 0.06 a	0.96 ± 0.02 c	1.01 ± 0.12 c
Carotenoid content (mg/g DW)	0.14 ± 0.00 b	0.21 ± 0.01 a	0.17 ± 0.02 b	0.15 ± 0.02 b
Chlorophyll a/b	2.03 ± 0.49 b	2.79 ± 0.23 a	3.12 ± 0.13 a	2.69 ± 0.01 a
POD (Ug/min)	99.23 ± 5.53 bc	160.30 ± 39.71 a	67.56 ± 5.19 c	112.08 ± 16.08 b
SOD (U/g)	2452.34 ± 30.66 b	3350.91 ± 221.24 a	2229.87 ± 346.01 b	3197.89 ± 568.07 a
IAA (ng/g)	19.56 ± 0.95 a	14.57 ± 0.51 b	10.41 ± 0.58 c	15.04 ± 0.05 b
GA (ng/g)	312.09 ± 0.99 a	260.51 ± 0.47 b	208.12 ± 0.90 d	219.66 ± 0.57 c

*L1 and L2 denote the two light intensities; Tri and NTri denote the treatment groups inoculated and not inoculated with Trichoderma, respectively.

Lowercase letters a, b, c, and d in the table denote the statistical significance between different treatment combinations for the parameter on the row.

Notable interactive effects of changes in light intensity and *Trichoderma* inoculation on plant physiological traits were observed. As shown in **Table 2**, when the categorical variable of different light intensities (L), the variable of inoculation (T), and the interaction (L×T) showed significance, it explained that *Trichoderma* inoculation had inconsistent effects on the physiological traits under different light intensities. These traits were manifested in the main parameters related to plant growth, including plant height, dry weight, plant N and P, chlorophyll, and GA, indicating interactions between L and T. In addition, it was noted that changing light intensity and *Trichoderma* inoculation affected leaf and stem development to varying degrees, significantly altered soil N, P content, and soil pH, and further regulated the activity of peroxidase (POD) and superoxide dismutase (SOD) in plants. Since the changes in these traits were not significant as L×T, it indicates that inoculation had significant and consistent effects under different light treatments.

**Table 2:** Two-way analysis of variance indicating the interactive effects of independent variables (light supply and *Trichoderma* inoculation treatment) and their co-influence on dependent variables.

Physiological traits		Treatment combinations^†^		
		L	T	L×T
Plant height		**	**	**
Stem diameter		**	**	n.s.
Leaf area		n.s.	**	n.s.
SLA		**	n.s.	n.s.
Shoot dry weight		**	**	**
Root dry weight		**	**	*
Total dry weight		**	**	**
Shoot/Root		**	**	**
Plant N		**	**	**
Plant P		**	**	**
Soil N		**	*	n.s.
Soil P		**	*	n.s.
Soil pH		n.s.	**	*
Chlorophyll a		**	**	**
Chlorophyll b		**	n.s.	n.s.
Total chlorophyll content		**	**	*
Carotenoid content		*	*	**
Chlorophyll a/b		*	n.s.	**
POD activity		*	**	n.s.
SOD activity		n.s.	**	n.s.
IAA content		**	n.s.	**
GA content		**	**	**

^†^L represents the categorical variable of different light intensities, T represents the categorical variable of whether applied Trichoderma inoculation or not, L×T represents the interaction of the two variables.

* and ** indicate that the interaction between these terms was significant at p=0.05 and p=0.01, respectively.

n.s. means that no significance was found.

### Antioxidant enzymes activities and phytohormone contents

The POD and SOD activity was significantly (p<0.05) influenced by light conditions and *Trichoderma* inoculation. The POD and SOD for the Tri group under L1 were 1.62 and 1.37 times higher than the NTri group, while The POD and SOD for the Tri group under L2 were 1.66 and 1.43 times higher than the NTri group (**Table 1**). The results showed that the activities of antioxidant enzymes in the plants of each treatment group were ranked in descending order of L2 + Tri > L1 + Tri > L1 + NTri > L2 + NTri. The contents of Indole-3-acetic acid (IAA) and gibberellic acid (GA) were significantly (p<0.05) influenced by light conditions and *Trichoderma* inoculation. Under the L1 condition, the highest IAA and GA contents were observed in the NTri group (**Table 1**). In the L1 condition, the IAA and GA contents for the Tri group were 0.78 and 0.52 times lower than the NTri group, respectively. In the L2 condition, the IAA content for the Tri group was 1.48 times higher than the NTri group.

### Interactive effects of the Trichoderma inoculation and artificial light supply on plant growth

**Figure 3:**
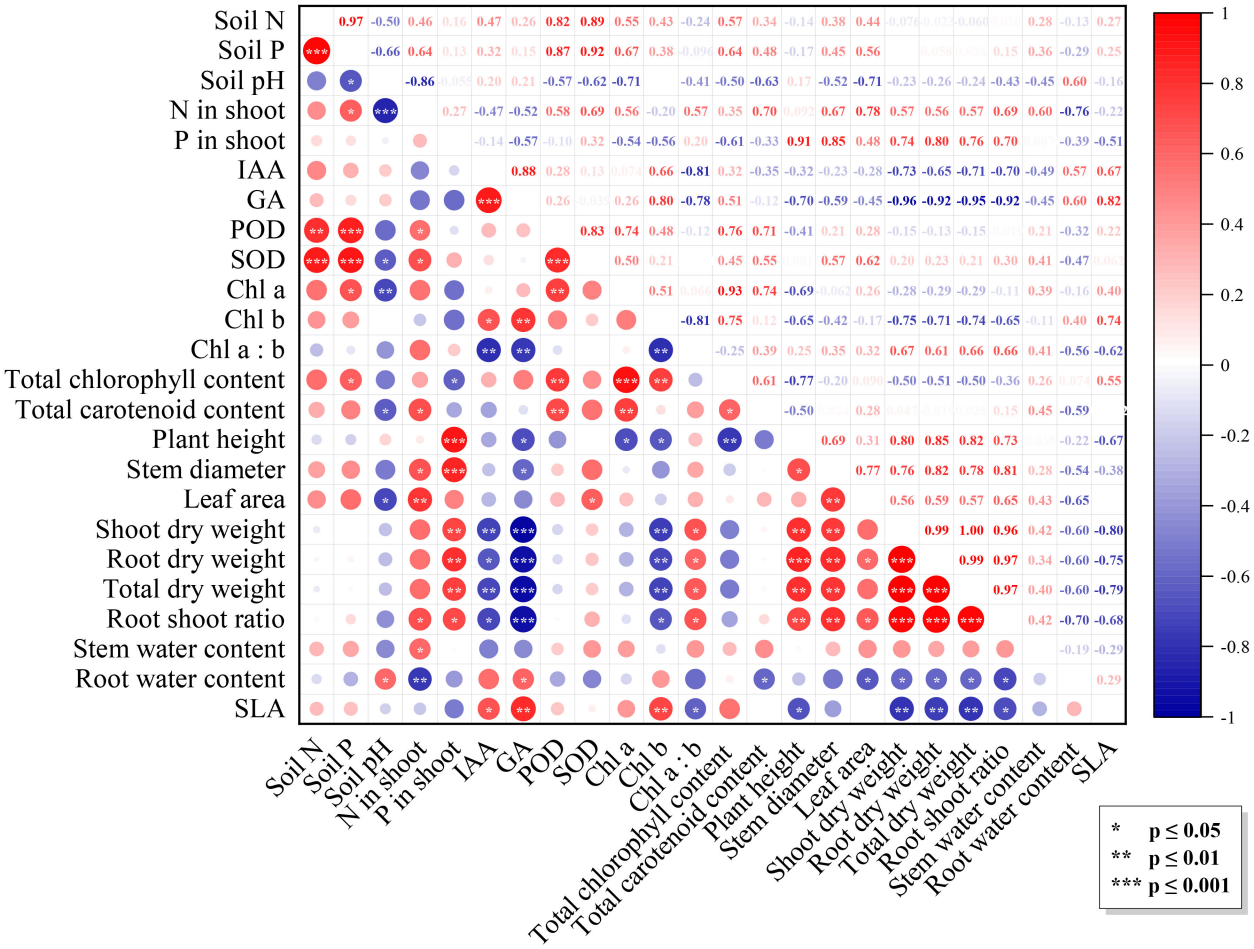
Correlation matrix of the factors. The upper triangular part of the graph is the correlation coefficient matrix, the lower triangular part is the graphical indication, with the diameter of the dots denoting the values of the correlation coefficients: positive and negative correlations are indicated by colors, and significance levels are indicated by asterisks.

Multiple factors influenced *N. benthamiana* growth under the combined effects of *Trichoderma* inoculation and varying light intensity. **Figure 3** showed significant correlations among growth-related indicators (p ≤ 0.01), showing a red triangle in the correlation matrix. Meanwhile, these indicators were positively correlated with N and P content in plants (p ≤ 0.01) and significantly negatively correlated with IAA, GA, and Chl a (p ≤ 0.01). Activities of SOD and POD were correlated with the N and P concentrations in soil (p ≤ 0.01). Soil pH was negatively correlated with other indicators, especially with N content in the shoot (p ≤ 0.001), Chl a (p ≤ 0.01), SOD activity, carotenoid, and leaf area (p ≤ 0.05).

**Figure 4:**
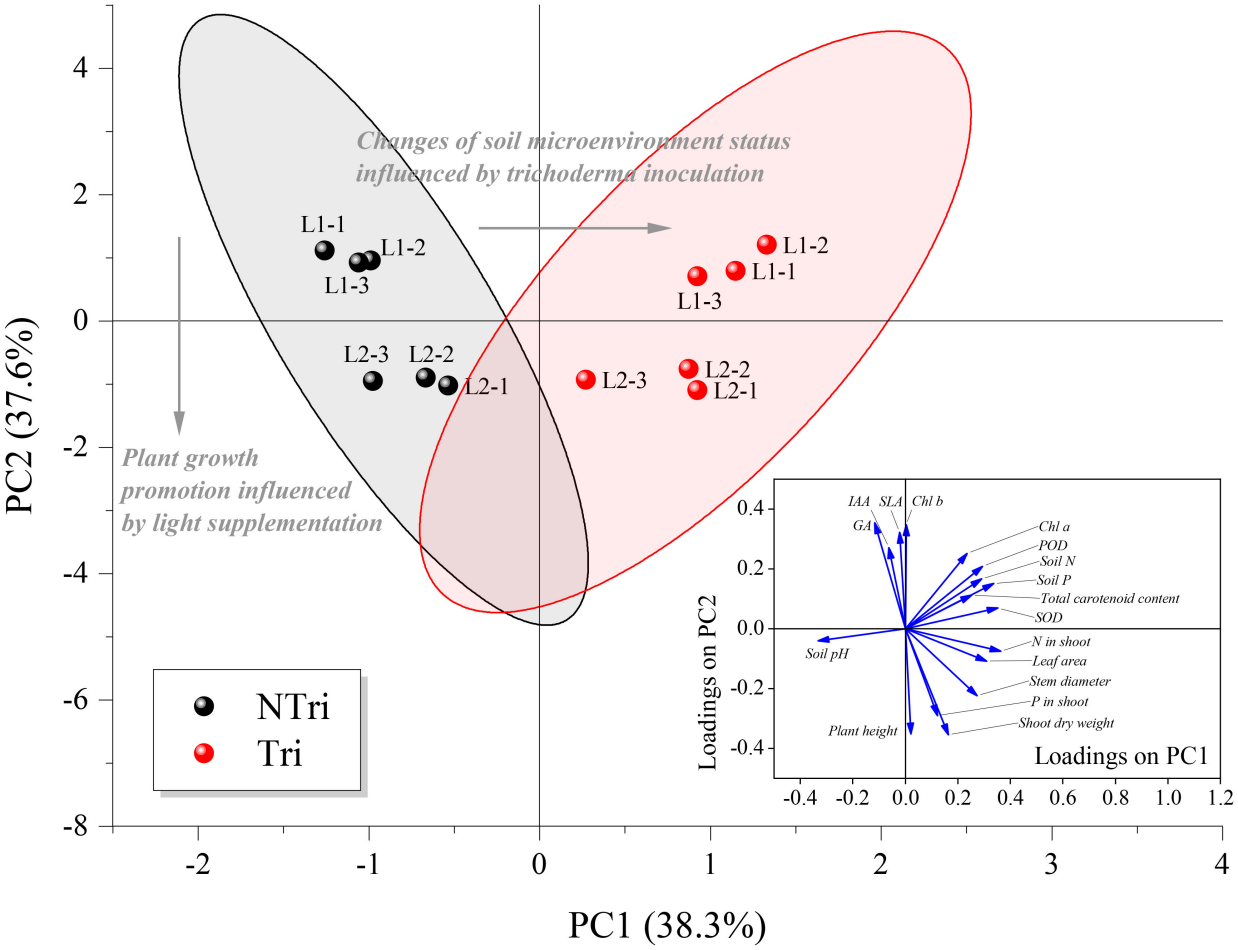
Biplot of principal component analysis (PCA) for the factors. NTri and Tri denote the Non-*Trichoderma* inoculated and *Trichoderma* inoculated groups, respectively. L1 and L2 denote the treatment groups with the light intensity of 50 and 150 μmol·m^-2^·s^-1^, respectively. Blue arrows denote the loadings, which are the coefficients of the linear combination of the factors that constitute the PCs. The gray arrows denote the principal influencing processes characterized by the sample scatter distribution. Samples of each treatment group are clustered and enveloped in their respective 95% confidence ellipse.

As shown in **Figure 4**, each treatment group was closely clustered, indicating that variation of light conditions and *Trichoderma* inoculation were statistically significant for clustering the data. The eigenvalues for principal component numbers 1 and 2 (PC1 and PC2) were 6.52 and 6.39, respectively. Their cumulative percentage of variance accounted for 75.89%. The loading scores of soil N and P concentrations, soil pH, plant leaf area, Chl a, carotenoid, N concentration in the shoot, and relevant enzyme activities (POD and SOD) were relatively high on PC1, ranging from 0.25 to 0.36; whereas for PC2, plant height, shoot dry weight, SLA, P in the shoot, GA, IAA, and Chl b concentrations had higher loading scores, ranging from 0.27 to 0.35. Thus, we determined that the loading scores for PC1 were mainly related to plant growth promotion influenced by light supplementation, while PC2 was particularly associated with changes in soil microenvironment status influenced by *Trichoderma* inoculation. It was apparent that *Trichoderma* inoculation separated the NTri and Tri groups from left to right on the PC1 axis, and different light treatments separated the L1 and L2 groups from top to bottom on the PC2 axis. Note that the L1 was positioned above the L2 on the PC2 axis within Tri or NTri treatment group. Concerning PC2, the plant height, shoot dry weight, and P in the shoot were negatively correlated with SLA, Chl b, and the contents of IAA and GA, indicating the effect of light on chlorophyll contents, phytohormone regulation, and growth of *N. benthamiana*. With the *Trichoderma* inoculation, the cluster shifted to the right; on the other hand, soil N and P content were correlated with N in the shoot, POD, SOD, and carotenoid, which could be explained by the fact that *Trichoderma* changed the soil environment, and thus affected the nutrient uptake and antioxidant levels of *N. benthamiana*.

## Discussion

### Mutualistic and parasitic interactions

It was worth noting that *Trichoderma* inoculation combined with two light conditions showed different effects (**Table 2**), reflecting that the beneficial effect of *Trichoderma* on plants must be based on a certain level of light energy conversion and utilization. The situation differed in the L1 condition, where plant height decreased after *Trichoderma* inoculation (**Figure 2**). When *Trichoderma* inoculation was applied in limited light conditions, the fungi were suggested to act as parasites and inhibited the growth of host plants. This parasitic interaction phenomenon is vital in symbionts’ development, especially under variable environmental conditions (Kiers et al., 2016). The conversion of light energy and its transfer process to the rhizosphere was the basis for influencing the *Trichoderma* colonization and proliferation. In this experiment, it was suggested that the transfer and exchange of resources between the aboveground parts of the plant and the rhizosphere and the metabolism of *Trichoderma* were the key processes influencing parasitic interactions.

Dry weight and root length were related to the proportion of allocation of the photosynthetic organic carbon (Andrews et al., 1999). However, reduced light intensity was detrimental to the accumulation of photosynthetic products and limited the development of the plant roots. When the light was insufficient, carbon tended to allocate towards stem elongation at the expense of root and leaf development, thus impairing the mass transfer to the rhizosphere (Gommers et al., 2013). As shown in **Figure 2**, plant dry weight decreased, and SLA increased as the light intensity declined. As this change facilitated an increase in light interception by leaves (Wu et al., 2017), it was usually manifested as thinner epidermal cells and fewer palisade tissue layers in leaves. The rate of anticlinal cell expansion and palisade cell layers declined, leading to thinner leaves and larger leaf area (Valladares and Niinemets, 2008; Terashima et al., 2011; Kalve et al., 2014). Note that this change in leaf morphology favors light interception, as Evans and Poorter (2001) showed that acclimation to low light resulted in a doubling of SLA and halving of organic nitrogen content per unit leaf area; however, it is detrimental to CO_2_ transport and photosynthesis, which ultimately affect biomass accumulation (Terashima et al., 2001).

From the perspective of the rhizosphere microenvironment, reproduction and metabolism of *Trichoderma* were indirectly affected by the influence of light on the aboveground part of the plant, which further affected the nutrient supply and growth of the plant. It was deduced that *Trichoderma* inoculation contributed to the absorption of N and P, thus improving chlorophyll synthesis in plants, as demonstrated by Elkelish et al. (2020) and Vukelić et al. (2021). *Trichoderma* may promote the solubilization of complex compounds by stimulating the secretion of organic acids from the roots (Zhang et al., 2014), and as a result, the soil pH was significantly reduced (**Table 1**). Concordant results were exhibited in Zhai et al. (2019) and El-Katatny (2010). These organic acids played a role in converting organic N to inorganic N and releasing modest amounts of NO_2_. Meanwhile, *Trichoderma* produced phosphatases, which converted the soil phosphate into di- or monobasic phosphates. Eventually, these phosphates were available for absorption (Maeda et al., 2015; Zhao et al., 2017).

Under L2 conditions, *Trichoderma* inoculation increased N and P content in plant tissues by 14% to 65% and improved plant height, leaf area, and dry weight (**Figure 2** and **Table 2**). However, as shown in **Figure 4**, light decreasing led to an upward scattering of the points on the PCA biplot, and the loading scores of Chl b were higher than Chl a on the PC2 axis. As Chl a/b decreased with light decreased, it could be due to the promotion of transformation from Chl a to Chl b. Also, the ratio of Chl a/b variation was indicative of whether the plants acclimate to low N availability (Kitajima and Hogan, 2003). The N supply to plants *via Trichoderma* could therefore be inhibited under low light conditions, which may contribute to the formation of the *Trichoderma* parasitic trait.

Compared with the NTri group, Tri group had a higher chlorophyll content, which may have facilitated plant photosynthesis, as the rate of photosynthetic was found to be highly correlated with the chlorophyll content (Buttery and Buzzell, 1977). Harman et al. (2021) indicated that *Trichoderma* inoculation induced up-regulation of relevant genes and pigments, which activated biochemical pathways that reduced reactive oxygen species (ROS) to less harmful molecules, thus improving photosynthesis in stressed plants. As revealed by the present study, the mutualistic and parasitic interaction phenomenon of *Trichoderma* is exhibited by nutrient uptake regulations and photosynthetic pigment synthesis.

### Regulation of phytohormones

In the application of PGPF, the coordination between light and strain proliferation was present, and the regulation of phytohormones was of central importance, including Indole-3-acetic acid (IAA) and gibberellic acid (GA). The signaling network of phytohormones is regulated and coordinated with light intensities through extensive receptors, signal transducers, and effector proteins (Franklin, 2008; Vaishak et al., 2019). IAA and GA were key phytohormones to regulate leaf development, expansion, elongation, and their contents determined their effects (positive or negative) on plant growth (Bhalerao et al., 2002; Gupta and Chakrabarty, 2014; Nieto-Jacobo et al., 2017). *Trichoderma* was involved in regulating the IAA and GA, which significantly modified the plant growth (Zhang et al., 2013; Ahmad et al., 2015). In the NTri group of this study, a reduction in plant height and stem diameter was found owing to the reduction in light and thus a deficit in photosynthetic energy supply, while high phytohormone concentrations inhibited cell expansion and division (**Table 1**) (Yang et al., 2014).

The IAA and GA content of the Tri group were lower than those of the NTri group under L1 conditions (**Table 1**). Related studies have shown that *Trichoderma* could regulate ethylene concentration by producing aminocyclopropane-1-carboxylic acid deaminase, reducing IAA and GA (Chowdappa et al., 2013; Hermosa et al., 2013; Kamalov et al., 2018; Jaroszuk-Sciseł et al., 2019). However, in the present study, the IAA contents of the Tri group were significantly higher than those of the NTri group under L2 conditions (**Table 1**), and the results were consistent with the previous study (Martínez-Medina et al., 2011). This phenomenon could be due to increased light intensity, rendering sufficient rhizodeposition from the adequate photosynthetic output (Harman, 2000). As a result, the activity of the *Trichoderma* was boosted, and its metabolites (e.g., Indole-3-pyruvic acid, Indole-3-acetaldehyde, and Tryptophan) regulated the phytohormone concentrations (Contreras-Cornejo et al., 2009; Sofo et al., 2011; Won et al., 2011). As confirmed by **Figure 3**, IAA and GA showed a significant positive correlation with plant Chl b with SLA. However, conversely, they were weakly or negatively correlated with plant growth, suggesting that the parasitism of *Trichoderma* may result in a reduction in symbiont vigor and that phytohormones did not solely stimulate plant growth. Evidence indicated that although *Trichoderma* could produce IAA and other diffusible compounds, their production was strain dependent and could be influenced by various external stimuli. Hence the phytostimulation may not solely or completely be interpreted by an auxinic mechanism (Nieto-Jacobo et al., 2017; Pelagio-Flores et al., 2017).

### Antioxidant activity

Insufficient light condition and *Trichoderma* proliferation are severe abiotic and biotic stresses for plants. The antioxidant activity of the symbionts may be critical in evaluating growth systems when coordinating the variable light intensity and *Trichoderma* inoculation. Plant tolerance under abiotic stresses was regulated by antioxidant defense systems (Bernal-Vicente et al., 2015). These antioxidant enzymes acted as “crosstalking” signals that coordinated the activity of defense networks complementary to the whole antioxidant system (Bernal-Vicente et al., 2015).

As shown in **Figure 4**, the activities of superoxide dismutase (SOD) and peroxidase (POD) accounted for the high loadings on PC1, which was interpreted as the causative factor of enzymatic antioxidants inducing the migration of NTri clusters on the PC1 axis. As confirmed in **Table 1**, *Trichoderma* inoculation was conducive to enhancing the activities of SOD and POD, which were positively correlated with total chlorophyll content, and similar results were reported in previous research (Gajera et al., 2016; Sarangi et al., 2021). Excessive accumulation of ROS in plants caused lipid peroxidation and ultimately damaged the plant cells (Ahmad et al., 2010; Gill and Tuteja, 2010; Soares et al., 2016). Antioxidant enzymes (including SOD and POD) were conducive to scavenging the ROS, and the activity of these enzymes reflected the level of oxidation resistance (Kapoor et al., 2019). The activities of SOD and POD of NTri groups were low compared with Tri groups (**Table 1**). The result revealed the existence of oxidative stress in plants, but the ability to mitigate ROS was limited. This phenomenon was previously confirmed in *Brassica* (Zhu et al., 2017), *Anoectochilus roxburghii* (Shao et al., 2014), and soybean plants (Raza et al., 2020). ROS production under low light stress could be due to the enhanced photorespiration accompanied by the inhibition of the synthesis of active molecules that scavenge the ROS (Blokhina, 2003; Chaki et al., 2009; Zhao, 2014).

Notably, the driving factor for the changes in antioxidant activities could be the alterations in soil microenvironment status (Wu et al., 2022), including changes in soil pH and C, N, and P contents. According to the results shown in **Figure 4**, the migration of cluster L1 + Tri to L2 + Tri reflected the mutualistic traits of *Trichoderma*, leading to an analogical result of plant growth promotion as light supplementation. The fitness of inoculated fungus always depends on plant carbon because they have no independent means of taking up carbon (Johnson, 2010; Grman, 2012). Higher light intensities ensured adequate rhizodeposition, reflected in alterations in the rhizosphere microenvironment and antioxidant activities, which eventually led to the mutualism-parasitism transition.

**Figure 5:**
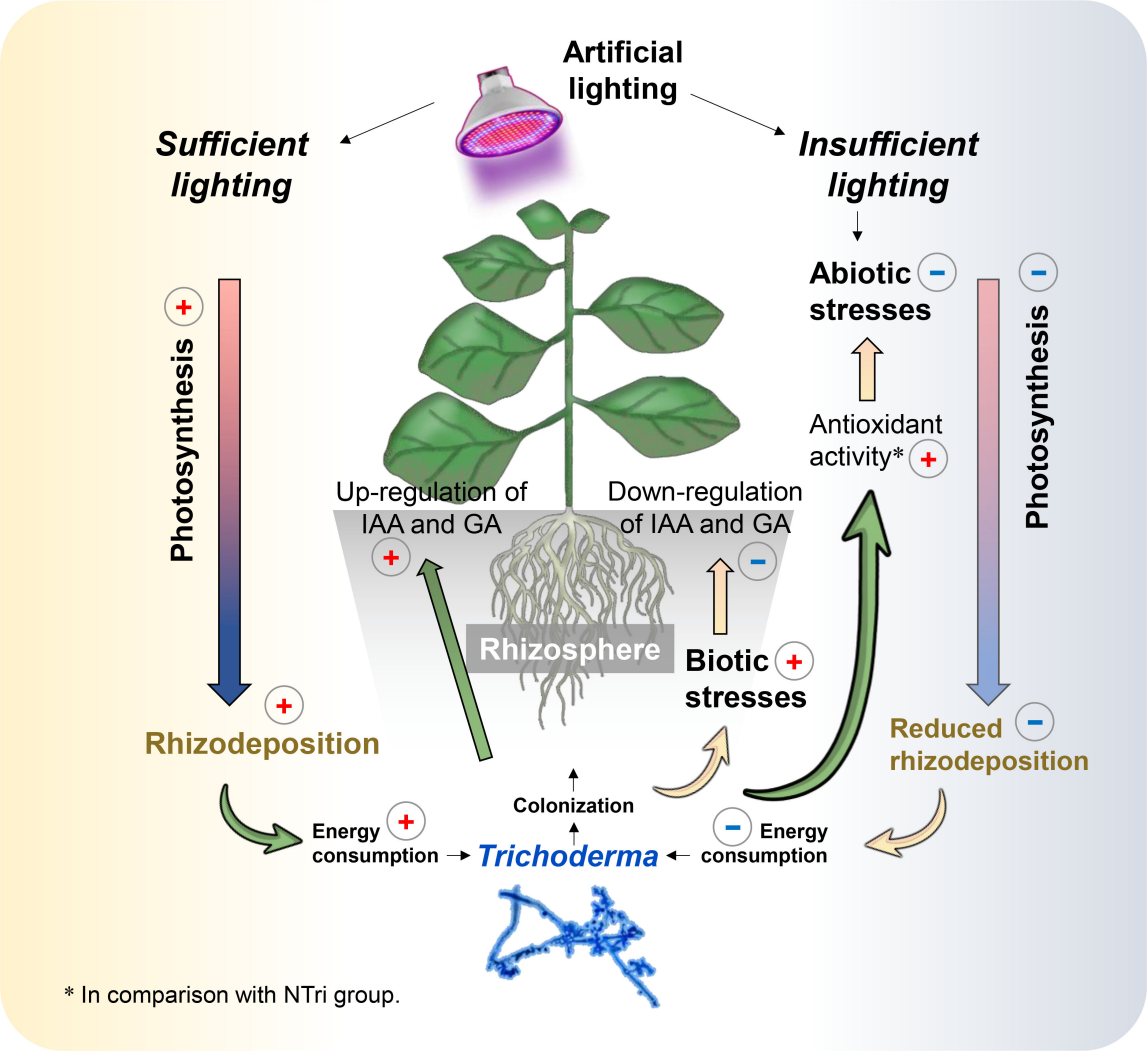
Schematic diagram of the interaction mechanism between artificial light supply and *Trichoderma* inoculation revealed in this study. Arrows indicate influence processes, positive red signs represent increase or enhancement, and negative blue signs represent reduction.

Based on the above analysis, the interaction between artificial light supply and *Trichoderma* inoculation on *N. benthamiana* was proposed, as illustrated in **Figure 5**. The effect of light changes on plants, including the content of Chl a, b and carotenoids, significantly contributed to rhizodeposition and energy consumption by *Trichoderma*. Furthermore, as adequate rhizodeposition boosted the *Trichoderma* activity and regulated the metabolites, it subsequently affected the phytohormone secretion of the plant, including IAA and GA. In addition, the antioxidant system of the symbiosis was sensitive to the light intensity and the colonization response of *Trichoderma*, which may be systematically indicative of the combination of growth parameters. The dynamics of C, N, and P in rhizodeposition exchanges between the plants and the *Trichoderma* could be explained as a biological market, in which these sources were reciprocally exchanged, with evolutions of resource exchange mutualisms (Konvalinková and Jansa, 2016; Afkhami et al., 2020). The rhizosphere microenvironment could be significantly altered during *Trichoderma* colonization, proliferation, and controlled light conditions. The rhizodeposition was a privileged and specialized resource for *Trichoderma*, especially in CEA models similar to this study that cultivate plants in the limited volume of a substrate cube (Tan et al., 2021).

## Conclusions and perspectives

This study conducted pot experiments to investigate the effect of *Trichoderma* inoculation on the growth, physiological, and biochemical traits of *N. benthamiana* under different light intensities. Succinctly, in high light conditions, inoculated *Trichoderma* participated in the mediation of phytohormones, improved antioxidant enzyme activity, and further increased nutrient uptake, thus promoting plant growth. In contrast, low light negatively affected plants by inducing oxidative impairment and inhibiting nutrient uptake, photosynthetic pigments accumulation, and phytohormone secretion. Low light stress could be alleviated by *Trichoderma* inoculation, which was beneficial in restoring the content of photosynthetic pigments and antioxidant enzymes. Nevertheless, in low light conditions, the promoting effect of *Trichoderma* on plant growth was limited due to insufficient rhizodeposition from the restricted photosynthetic output, rendering a transition of mutualism to parasitism during this process. We hope that the findings in this study can aid in increasing our understanding of the physiology of the symbiont under artificial light supply to optimize lighting and inoculation techniques on indoor farming.

In the context of precise regulation of the plant growth environment by CEA systems, multiple functions of biocontrol of *Trichoderma* are valuable to be explored and utilized, including the antibiosis, mycoparasitism, solubilization and sequestration of inorganic nutrients, induced resistance, inactivation of the pathogen’s enzymes and so on. However, the mechanisms of different species of *Trichoderma* spp. in functioning as plant growth promoters and biocontrol agents need to be further revealed, especially in the artificial growing environment. Given the complexities of the interactive effects of the variable light intensity and *Trichoderma* inoculation on growth, explicit consideration of symbiotic exchange for resources in soil- and substrate-based controlled environment agriculture is of central importance. Notably, the transition from mutualism to parasitism induced by resource trade competition during energy conservation in artificial light supply requires integration into the biological market theory. In subsequent studies, it is essential to understand how rhizodeposition modulates the outcome of *Trichoderma* symbiosis from a molecular biology perspective. Genes regulation and their products involved in the interaction of *Trichoderma* with plants are considered major issues that require further research intensively in CEA development.

## Data availability statement

The raw data supporting the conclusions of this article will be made available by the authors, without undue reservation.

## Author contributions

Conceptualization: BT and DD. Methodology: BT, YL, and HP. Material preparation, data collection and analysis: BT, YL, and YZ. Formal analysis and investigation: YL. Writing: YL, BT. Funding acquisition: BT, XT, and ZL. Supervision: WZ and BT. All authors read and approved the final manuscript.

## Acknowledgments

This study was funded by the Natural Science Foundation of Sichuan Province (2022NSFSC1654, 2022NSFSC0086), the Fundamental Research Funds for the Central Universities (2021SCU12037), the Key Research and Development Program of Sichuan Province, China (2019YFN0153). The authors would like to thank Ms. Huiting Xu from Sichuan Agricultural University for her help in isolating and purifying the fungi strain, and Ms. Sha Feng for her advice on polishing the artwork graphs and figures.

## Conflict of interest

Author YL is employed by Sichuan Development Guorun Water Investment Co. Ltd.

The remaining authors declare that the research was conducted in the absence of any commercial or financial relationships that could be construed as a potential conflict of interest.

## Publisher’s note

All claims expressed in this article are solely those of the authors and do not necessarily represent those of their affiliated organizations, or those of the publisher, the editors and the reviewers. Any product that may be evaluated in this article, or claim that may be made by its manufacturer, is not guaranteed or endorsed by the publisher.

## References

[B1] Afkhami M. E. Almeida B. K. Hernandez D. J. Kiesewetter K. N. Revillini D. P. (2020). Tripartite mutualisms as models for understanding plant–microbial interactions. Curr. Opin. Plant Biol. 56, 28–36. doi: 10.1016/j.pbi.2020.02.003 32247158

[B2] Ahmad P. Hashem A. Abd-Allah E. F. Alqarawi A. A. John R. Egamberdieva D.. (2015). Role of trichoderma harzianum in mitigating NaCl stress in Indian mustard (Brassica juncea l) through antioxidative defense system. Front. Plant Sci. 6. doi: 10.3389/fpls.2015.00868 PMC4604702 26528324

[B3] Ahmad P. Jaleel C. A. Salem M. A. Nabi G. Sharma S. (2010). Roles of enzymatic and nonenzymatic antioxidants in plants during abiotic stress. Crit. Rev. Biotechnol. 30, 161–175. doi: 10.3109/07388550903524243 20214435

[B4] Andrews M. Sprent J. I. Raven J. A. Eady P. E. (1999). Relationships between shoot to root ratio, growth and leaf soluble protein concentration of pisum sativum, phaseolus vulgaris and triticum aestivum under different nutrient deficiencies. Plant Cell Environ. 22, 949–958. doi: 10.1046/j.1365-3040.1999.00452.x

[B5] Bao S. (2000). Soil agricultural chemical analysis (Beijing: China Agricultural Press), 176–185.

[B6] Benke K. Tomkins B. (2017). Future food-production systems: Vertical farming and controlled-environment agriculture. Sustainability: Science Pract. Policy 13, 13–26. doi: 10.1080/15487733.2017.1394054

[B7] Berg G. (2009). Plant-microbe interactions promoting plant growth and health: Perspectives for controlled use of microorganisms in agriculture. Appl. Microbiol. Biot. 84, 11–18. doi: 10.1007/s00253-009-2092-7 19568745

[B8] Bernal-Vicente A. Pascual J. A. Tittarelli F. Hernández J. A. Diaz-Vivancos P. (2015). Trichoderma harzianum T-78 supplementation of compost stimulates the antioxidant defence system in melon plants. J. Sci. Food Agr. 95, 2208–2214. doi: 10.1002/jsfa.6936 25255983

[B9] Bhalerao R. P. Eklöf J. Ljung K. Marchant A. Bennett M. Sandberg G. (2002). Shoot-derived auxin is essential for early lateral root emergence in arabidopsis seedlings. Plant J. 29, 325–332. doi: 10.1046/j.0960-7412.2001.01217.x 11844109

[B10] Blokhina O. (2003). Antioxidants, oxidative damage and oxygen deprivation stress: a review. Ann. Bot.-London 91, 179–194. doi: 10.1093/aob/mcf118 PMC4244988 12509339

[B11] Blokhina O. Virolainen E. Fagerstedt K. V. (2003). Antioxidants, oxidative damage and oxygen deprivation stress: a review. Ann. Bot.-London 91, 179–194. doi: 10.1093/aob/mcf118 PMC4244988 12509339

[B12] Bononi L. Chiaramonte J. B. Pansa C. C. Moitinho M. A. Melo I. S. (2020). Phosphorus-solubilizing trichoderma spp. from Amazon soils improve soybean plant growth. Sci. Rep.-UK 10. doi: 10.1038/s41598-020-59793-8 PMC7028723 32071331

[B13] Buttery B. R. Buzzell R. I. (1977). Relationship between chlorophyll content and rate of photosynthesis in soybeans. Can. J. Plant Sci. 57, 1–5. doi: 10.4141/cjps77-001

[B14] Carvalho L. C. Santos S. Jorge Vilela B. Amâncio S. (2008). Solanum lycopersicon mill. and nicotiana benthamiana l. under high light show distinct responses to anti-oxidative stress. J. Plant Physiol. 165, 1300–1312. doi: 10.1016/j.jplph.2007.04.009 17945381

[B15] Chaki M. Fernández-Ocaña A. M. Valderrama R. Carreras A. Esteban F. J. Luque F.. (2009). Involvement of reactive nitrogen and oxygen species (RNS and ROS) in sunflower–mildew interaction. Plant Cell Physiol. 50, 265–279. doi: 10.1093/pcp/pcn196 19112080

[B16] Chet I. Harman G. E. Baker R. (1981). Trichoderma hamatum: Its hyphal interactions with rhizoctonia solani and pythium spp. Microb. Ecol. 7, 29–38. doi: 10.1007/BF02010476 24227317

[B17] Chowdappa P. Mohan Kumar S. P. Jyothi Lakshmi M. Upreti K. K. (2013). Growth stimulation and induction of systemic resistance in tomato against early and late blight by bacillus subtilis OTPB1 or trichoderma harzianum OTPB3. Biol. Control 65, 109–117. doi: 10.1016/j.biocontrol.2012.11.009

[B18] Contreras-Cornejo H. A. Macías-Rodríguez L. Cortés-Penagos C. López-Bucio J. (2009). Trichoderma virens, a plant beneficial fungus, enhances biomass production and promotes lateral root growth through an auxin-dependent mechanism in arabidopsis. Plant Physiol. 149, 1579–1592. doi: 10.1104/pp.108.130369 19176721 PMC2649400

[B19] Cortés C. Gutiérrez A. Olmedo V. Inbar J. Chet I. Herrera-Estrella A. (1998). The expression of genes involved in parasitism by trichoderma harzianum is triggered by a diffusible factor. Mol. Gen. Genet. MGG 260, 218–225. doi: 10.1007/s004380050889 9862475

[B20] Das K. Roychoudhury A. (2014). Reactive oxygen species (ROS) and response of antioxidants as ROS-scavengers during environmental stress in plants. Front. Environ. Sci. 2. doi: 10.3389/fenvs.2014.00053

[B21] De Jaeger N. de la Providencia I. E. Dupré De Boulois H. Declerck S. (2011). Trichoderma harzianum might impact phosphorus transport by arbuscular mycorrhizal fungi. FEMS Microbiol. Ecol. 77, 558–567. doi: 10.1111/j.1574-6941.2011.01135.x 21609342

[B22] El-Katatny M. H. (2010). Enzyme production and nitrogen fixation by free, immobilized and coimmobilized inoculants of trichoderma harzianum and azospirillum brasilense and their possible role in growth promotion of tomato. Food Technol. Biotech. 48, 161–174. doi: 10.1016/j.fm.2009.10.014

[B23] Elkelish A. A. Alhaithloul H. A. S. Qari S. H. Soliman M. H. Hasanuzzaman M. (2020). Pretreatment with trichoderma harzianum alleviates waterlogging-induced growth alterations in tomato seedlings by modulating physiological, biochemical, and molecular mechanisms. Environ. Exp. Bot. 171, 103946. doi: 10.1016/j.envexpbot.2019.103946

[B24] Engler N. Krarti M. (2021). Review of energy efficiency in controlled environment agriculture. Renew. Sust. Energ. Rev. 141. doi: 10.1016/j.rser.2021.110786

[B25] Evans J. R. Poorter H. (2001). Photosynthetic acclimation of plants to growth irradiance: the relative importance of specific leaf area and nitrogen partitioning in maximizing carbon gain. Plant Cell Environ. 24, 755–767. doi: 10.1046/j.1365-3040.2001.00724.x

[B26] Farhangi M. H. Turvani M. E. van der Valk A. Carsjens G. J. (2020). High-tech urban agriculture in Amsterdam: An actor network analysis. Sustainability-Basel 12, 3955. doi: 10.3390/su12103955

[B27] Feng L. Raza M. A. Li Z. Chen Y. Khalid M. H. B. Du J.. (2019). The influence of light intensity and leaf movement on photosynthesis characteristics and carbon balance of soybean. Front. Plant Sci. 9. doi: 10.3389/fpls.2018.01952 PMC6338029 30687355

[B28] Franklin K. A. (2008). Shade avoidance. New Phytol. 179, 930–944. doi: 10.1111/j.1469-8137.2008.02507.x 18537892

[B29] Gajera H. P. Katakpara Z. A. Patel S. V. Golakiya B. A. (2016). Antioxidant defense response induced by trichoderma viride against aspergillus niger van tieghem causing collar rot in groundnut (Arachis hypogaea l.). Microb. Pathogenesis 91, 26–34. doi: 10.1016/j.micpath.2015.11.010 26620080

[B30] Gama P. B. S. Tanaka K. Eneji A. E. Eltayeb A. E. Siddig K. E. (2009). Salt-induced stress effects on biomass, photosynthetic rate, and reactive oxygen species-scavenging enzyme accumulation in common bean. J. Plant Nutr. 32, 837–854. doi: 10.1080/01904160902787925

[B31] Gehring C. A. (2003). Growth responses to arbuscular mycorrhizae by rain forest seedlings vary with light intensity and tree species. Plant Ecol. 167, 127–139. doi: 10.1023/A:1023989610773

[B32] Gill S. S. Tuteja N. (2010). Reactive oxygen species and antioxidant machinery in abiotic stress tolerance in crop plants. Plant Physiol. Bioch. 48, 909–930. doi: 10.1016/j.plaphy.2010.08.016 20870416

[B33] Gommers C. M. M. Visser E. J. W. Onge K. R. S. Voesenek L. A. C. J. Pierik R. (2013). Shade tolerance: when growing tall is not an option. Trends Plant Sci. 18, 65–71. doi: 10.1016/j.tplants.2012.09.008 23084466

[B34] Goodin M. M. Zaitlin D. Naidu R. A. Lommel S. A. (2008). Nicotiana benthamiana: Its history and future as a model for plant-pathogen interactions. Mol. Plant Microbe in. 21, 1015–1026. doi: 10.1094/MPMI-21-8-1015 18616398

[B35] Grman E. (2012). Plant species differ in their ability to reduce allocation to non-beneficial arbuscular mycorrhizal fungi. Ecology 93, 711–718. doi: 10.1890/11-1358.1 22690621

[B36] Gupta R. Chakrabarty S. K. (2014). Gibberellic acid in plant. Plant Signaling Behav. 8, e25504. doi: 10.4161/psb.25504 PMC4002599 23857350

[B37] Guzmán-Guzmán P. Porras-Troncoso M. D. Olmedo-Monfil V. Herrera-Estrella A. (2019). Trichoderma species: Versatile plant symbionts. Phytopathology 109, 6–16. doi: 10.1094/PHYTO-07-18-0218-RVW 30412012

[B38] Harman G. E. (2000). Myths and dogmas of biocontrol changes in perceptions derived from research on trichoderma harzinum T-22. Plant Dis. 84, 377–393. doi: 10.1094/PDIS.2000.84.4.377 30841158

[B39] Harman G. E. (2011). Multifunctional fungal plant symbionts: new tools to enhance plant growth and productivity. New Phytol. 189, 647–649. doi: 10.1111/j.1469-8137.2010.03614.x 21223281

[B40] Harman G. E. Doni F. Khadka R. B. Uphoff N. (2021). Endophytic strains of trichoderma increase plants’ photosynthetic capability. J. Appl. Microbiol. 130, 529–546. 31271695 10.1111/jam.14368

[B41] Hazzoumi Z. Moustakime Y. Joutei K. A. (2014). Effect of gibberellic acid (GA), indole acetic acid (IAA) and benzylaminopurine (BAP) on the synthesis of essential oils and the isomerization of methyl chavicol and trans-anethole in ocimum gratissimum L. SPRINGERPLUS 3. doi: 10.1186/2193-1801-3-321 PMC4101128 25045609

[B42] Hermosa R. Rubio M. B. Cardoza R. E. Nicolas C. Monte E. Gutierrez S. (2013). The contribution of trichoderma to balancing the costs of plant growth and defense. Int. Microbiol. 16, 69–80. doi: 10.2436/20.1501.01.181 24400524

[B43] Hughes M. Donnelly C. Crozier A. Wheeler C. T. (1999). Effects of the exposure of roots of alnus glutinosa to light on flavonoids and nodulation. Can. J. botany-revue Can. botanique 77, 1311–1315. doi: 10.1139/b99-077

[B44] Illescas M. Pedrero-Méndez A. Pitorini-Bovolini M. Hermosa R. Monte E. (2021). Phytohormone production profiles in trichoderma species and their relationship to wheat plant responses to water stress. Pathogens 10, 991. doi: 10.3390/pathogens10080991 34451455 PMC8400765

[B45] Jaroszuk-Sciseł J. Tyśkiewicz R. Nowak A. Ozimek E. Majewska M. Hanaka A.. (2019). Phytohormones (Auxin, gibberellin) and ACC deaminase *In vitro* synthesized by the mycoparasitic trichoderma DEMTkZ3A0 strain and changes in the level of auxin and plant resistance markers in wheat seedlings inoculated with this strain conidia. Int. J. Mol. Sci. 20, 4923. doi: 10.3390/ijms20194923 PMC6801869 31590281

[B46] Johnson N. C. (2010). Resource stoichiometry elucidates the structure and function of arbuscular mycorrhizas across scales. New Phytol. 185, 631–647. doi: 10.1111/j.1469-8137.2009.03110.x 19968797

[B47] Kalantari F. Tahir O. M. Joni R. A. Fatemi E. (2018). Opportunities and challenges in sustainability of vertical farming: A review. J. Landscape Ecol. 11, 35–60. doi: 10.1515/jlecol-2017-0016

[B48] Kalve S. Fotschki J. Beeckman T. Vissenberg K. Beemster G. T. S. (2014). Three-dimensional patterns of cell division and expansion throughout the development of arabidopsis thaliana leaves. J. Exp. Bot. 65, 6385–6397. doi: 10.1093/jxb/eru358 25205574

[B49] Kamalov L. S. Turgunov K. K. Aripova S. F. Abdilalimov O. (2018). Gibberillin a-3 from the microscopic fungus trichoderma harzianum. Chem. Nat. Compd.+ 54, 421–422. doi: 10.1007/s10600-018-2368-1

[B50] Kapoor D. Singh S. Kumar V. Romero R. Prasad R. Singh J. (2019). Antioxidant enzymes regulation in plants in reference to reactive oxygen species (ROS) and reactive nitrogen species (RNS). Plant Gene 19, 100182. doi: 10.1016/j.plgene.2019.100182

[B51] Khalid A. Aftab F. (2020). Effect of exogenous application of IAA and GA3 on growth, protein content, and antioxidant enzymes of Solanum tuberosum L. grown in vitro under salt stress. In Vitro Cell. Dev.-Pl. 56, 377–389. doi: 10.1007/s11627-019-10047-x

[B52] Khoshmanzar E. Aliasgharzad N. Neyshabouri M. R. Khoshru B. Arzanlou M. Asgari Lajayer B. (2020). Effects of trichoderma isolates on tomato growth and inducing its tolerance to water-deficit stress. Int. J. Environ. Sci. Te. 17, 869–878. doi: 10.1007/s13762-019-02405-4

[B53] Kiers E. T. West S. A. Wyatt G. A. K. Gardner A. Bücking H. Werner G. D. A. (2016). Misconceptions on the application of biological market theory to the mycorrhizal symbiosis. Nat. Plants 2. doi: 10.1038/nplants.2016.63 27243656

[B54] Kitajima K. Hogan K. P. (2003). Increases of chlorophyll a/b ratios during acclimation of tropical woody seedlings to nitrogen limitation and high light. Plant Cell Environ. 26, 857–865. doi: 10.1046/j.1365-3040.2003.01017.x 12803613

[B55] Konvalinková T. Jansa J. (2016). Lights off for arbuscular mycorrhiza: On its symbiotic functioning under light deprivation. Front. Plant Sci. 7. doi: 10.3389/fpls.2016.00782 PMC4893486 27375642

[B56] KonvalinkovÃ T. Püchel D. JanouÅ KovÃ M. Gryndler M. Jansa J. (2015). Duration and intensity of shade differentially affects mycorrhizal growth- and phosphorus uptake responses of medicago truncatula. Front. Plant Sci. 6. doi: 10.3389/fpls.2015.00065 PMC4327418 25763002

[B57] Kotakis C. Vrettos N. Kotsis D. Tsagris M. Kotzabasis K. Kalantidis K. (2010). Light intensity affects RNA silencing of a transgene in nicotiana benthamiana plants. BMC Plant Biol. 10. doi: 10.1186/1471-2229-10-220 PMC3017829 20939918

[B58] Koubouris G. Bouranis D. Vogiatzis E. Nejad A. R. Giday H. Tsaniklidis G.. (2018). Leaf area estimation by considering leaf dimensions in olive tree. Sci. Hortic.-Amsterdam 240, 440–445. doi: 10.1016/j.scienta.2018.06.034

[B59] Kredics L. Naeimi S. Hatvani L. Vágvölgyi C. Cai F. Druzhinina I. S.. (2021). ‘The good, the bad and the ugly’ in the shades of green: the genus trichoderma in the spotlight. Indian Phytopathol. 74, 403–411. doi: 10.1007/s42360-021-00352-0

[B60] Lee S. Lee J. (2015). Beneficial bacteria and fungi in hydroponic systems: Types and characteristics of hydroponic food production methods. Sci. Hortic.-Amsterdam 195, 206–215. doi: 10.1016/j.scienta.2015.09.011

[B61] Li S. Tian Y. Wu K. Ye Y. Yu J. Zhang J.. (2018). Modulating plant growth-metabolism coordination for sustainable agriculture. Nature 560, 595–59+. doi: 10.1038/s41586-018-0415-5 30111841 PMC6155485

[B62] Maeda K. Spor A. Edel-Hermann V. Heraud C. Breuil M. Bizouard F.. (2015). N2O production, a widespread trait in fungi. Sci. Rep.-UK 5, 9697. doi: 10.1038/srep09697 PMC4403702 25894103

[B63] Mahato S. Neupane S. (2018). Comparative study of impact of azotobacter and trichoderma with other fertilizers on maize growth. J. Maize Res. Dev. 3, 1–16. doi: 10.3126/jmrd.v3i1.18915

[B64] Martínez-Medina A. Roldán A. Albacete A. Pérez-Alfocea F. Pascual J. A. (2011). Hormonal signalling of the trichoderma harzianum-induced resistance to fusarium oxysporum and growth promotion effect in melon plants. Acta Hortic. 898, 61–67. doi: 10.17660/ActaHortic.2011.898.6

[B65] Mittler R. Vanderauwera S. Gollery M. Van Breusegem F. (2004). Reactive oxygen gene network of plants. Trends Plant Sci. 9, 490–498. doi: 10.1016/j.tplants.2004.08.009 15465684

[B66] Moran-Diez M. E. Trushina N. Lamdan N. L. Rosenfelder L. Mukherjee P. K. Kenerley C. M.. (2015). Host-specific transcriptomic pattern of trichoderma virens during interaction with maize or tomato roots. BMC Genomics 16. doi: 10.1186/s12864-014-1208-3 PMC4326404 25608961

[B67] Moreira V. D. Oliveira C. Jalal A. Gato I. Oliveira T. Boleta G.. (2022). Inoculation with trichoderma harzianum and azospirillum brasilense increases nutrition and yield of hydroponic lettuce. Arch. Microbiol. 204. doi: 10.1007/s00203-022-03047-w 35771351

[B68] Nieto-Jacobo M. F. Steyaert J. M. Salazar-Badillo F. B. Nguyen D. V. Rostás M. Braithwaite M.. (2017). Environmental growth conditions of trichoderma spp. affects indole acetic acid derivatives, volatile organic compounds, and plant growth promotion. Front. Plant Sci. 8. doi: 10.3389/fpls.2017.00102 PMC5299017 28232840

[B69] Oliveira C. Jalal A. Oliveira J. R. Tamburi K. V. Teixeira M. (2022). Leaf inoculation of azospirillum brasilense and trichoderma harzianum in hydroponic arugula improve productive components and plant nutrition and reduce leaf nitrate. PESQUISA AGROPECUARIA Trop. 52. doi: 10.1590/1983-40632022v5272755

[B70] Paradiso R. Arena C. De Micco V. Giordano M. Aronne G. De Pascale S. (2017). Changes in leaf anatomical traits enhanced photosynthetic activity of soybean grown in hydroponics with plant growth-promoting microorganisms. Front. Plant Sci. 8. doi: 10.3389/fpls.2017.00674 PMC5418343 28529515

[B71] Pelagio-Flores R. Esparza-Reynoso S. Garnica-Vergara A. López-Bucio J. Herrera-Estrella A. (2017). Trichoderma-induced acidification is an early trigger for changes in arabidopsis root growth and determines fungal phytostimulation. Front. Plant Sci. 8. doi: 10.3389/fpls.2017.00822 PMC5434454 28567051

[B72] Pretty J. (2018). Intensification for redesigned and sustainable agricultural systems. Science 362, 908. doi: 10.1126/science.aav0294 30467142

[B73] Raza M. A. Feng L. Y. Iqbal N. Khan I. Meraj T. A. Xi Z. J.. (2020). Effects of contrasting shade treatments on the carbon production and antioxidant activities of soybean plants. Funct. Plant Biol. 47, 342. doi: 10.1071/FP19213 32040939

[B74] Sammauria R. Kumawat S. Kumawat P. Singh J. Jatwa T. K. (2020). Microbial inoculants: potential tool for sustainability of agricultural production systems. Arch. Microbiol. 202, 677–693. doi: 10.1007/s00203-019-01795-w 31897539

[B75] Sarangi S. Swain H. Adak T. Bhattacharyya P. Mukherjee A. K. Kumar G.. (2021). Trichoderma-mediated rice straw compost promotes plant growth and imparts stress tolerance. Environ. Sci. pollut. R 28, 44014–44027. doi: 10.1007/s11356-021-13701-3 33846916

[B76] Scandalios J. G. (1993). Oxygen stress and superoxide dismutases. Plant Physiol. 101, 7–12. doi: 10.1104/pp.101.1.7 12231660 PMC158641

[B77] Shao Q. Wang H. Guo H. Zhou A. Huang Y. Sun Y.. (2014). Effects of shade treatments on photosynthetic characteristics, chloroplast ultrastructure, and physiology of anoectochilus roxburghii. PLos One 9, e85996. doi: 10.1371/journal.pone.0085996 24516523 PMC3917826

[B78] Shoresh M. Harman G. E. Mastouri F. (2010). Induced systemic resistance and plant responses to fungal biocontrol agents. Annu. Rev. Phytopathol. 48, 21–43. doi: 10.1146/annurev-phyto-073009-114450 20192757

[B79] Singh B. N. Dwivedi P. Sarma B. K. Singh G. S. Singh H. B. (2019). A novel function of n-signaling in plants with special reference to trichoderma interaction influencing plant growth, nitrogen use efficiency, and cross talk with plant hormones. Biotech 3, 9. doi: 10.1007/s13205-019-1638-3 PMC6393646 30863693

[B80] Singh D. P. Singh V. Shukla R. Sahu P. Prabha R. Gupta A.. (2020). Stage-dependent concomitant microbial fortification improves soil nutrient status, plant growth, antioxidative defense system and gene expression in rice. Microbiol. Res. 239, 126538. doi: 10.1016/j.micres.2020.126538 32717536

[B81] Soares C. de Sousa A. Pinto A. Azenha M. Teixeira J. Azevedo R. A.. (2016). Effect of 24-epibrassinolide on ROS content, antioxidant system, lipid peroxidation and Ni uptake in solanum nigrum l. under Ni stress. Environ. Exp. Bot. 122, 115–125. doi: 10.1016/j.envexpbot.2015.09.010

[B82] Sofo A. Scopa A. Manfra M. De Nisco M. Tenore G. Troisi J.. (2011). Trichoderma harzianum strain T-22 induces changes in phytohormone levels in cherry rootstocks (Prunus cerasus × p. canescens). Plant Growth Regul. 65, 421–425. doi: 10.1007/s10725-011-9610-1

[B83] Tan B. Li Y. Liu T. Tan X. He Y. You X.. (2021). Response of plant rhizosphere microenvironment to water management in soil- and substrate-based controlled environment agriculture (CEA) systems: A review. Front. Plant Sci. 12. doi: 10.3389/fpls.2021.691651 PMC8385539 34456936

[B84] Tarroum M. Ben Romdhane W. Ali A. Al-Qurainy F. Al-Doss A. Fki L.. (2021). Harnessing the rhizosphere of the halophyte grass aeluropus littoralis for halophilic plant-Growth-Promoting fungi and evaluation of their biostimulant activities. PLANTS-BASEL 10. doi: 10.3390/plants10040784 PMC8073152 33923476

[B85] Terashima I. Hanba Y. T. Tholen D. Niinemets Ü. (2011). Leaf functional anatomy in relation to photosynthesis. Plant Physiol. 155, 108–116. doi: 10.1104/pp.110.165472 21075960 PMC3075775

[B86] Terashima I. Miyazawa S. Hanba Y. T. (2001). Why are sun leaves thicker than shade leaves? — consideration based on analyses of CO2 diffusion in the leaf. J. Plant Res. 114, 93–105. doi: 10.1007/PL00013972

[B87] Tester M. Smith F. A. Smith S. E. (1985). Phosphate inflow into trifolium subterraneum l.: Effects of photon irradiance and mycorrhizal infection. Soil Biol. Biochem. 17, 807–810. doi: 10.1016/0038-0717(85)90137-3

[B88] Triantaphylidès C. Krischke M. Hoeberichts F. A. Ksas B. Gresser G. Havaux M.. (2008). Singlet oxygen is the major reactive oxygen species involved in photooxidative damage to plants. Plant Physiol. 148, 960–968. doi: 10.1104/pp.108.125690 18676660 PMC2556806

[B89] Vaishak K. P. Yadukrishnan P. Bakshi S. Kushwaha A. K. Ramachandran H. Job N.. (2019). The b-box bridge between light and hormones in plants. J. Photochem. Photobiol. B: Biol. 191, 164–174. doi: 10.1016/j.jphotobiol.2018.12.021 30640143

[B90] Valladares F. Niinemets Ü. (2008). Shade tolerance, a key plant feature of complex nature and consequences. Annu. Rev. Ecology Evolution Systematics 39, 237–257. doi: 10.1146/annurev.ecolsys.39.110707.173506

[B91] Vukelić I. D. Prokić L. T. Racić G. M. Pešić M. B. Bojović M. M. Sierka E. M.. (2021). Effects of trichoderma harzianum on photosynthetic characteristics and fruit quality of tomato plants. Int. J. Mol. Sci 22, 6961. doi: 10.3390/ijms22136961 34203436 PMC8268988

[B92] Wipf D. Krajinski F. Tuinen D. Recorbet G. Courty P. E. (2019). Trading on the arbuscular mycorrhiza market: from arbuscules to common mycorrhizal networks. New Phytol. 223, 1127–1142. doi: 10.1111/nph.15775 30843207

[B93] Won C. Shen X. Mashiguchi K. Zheng Z. Dai X. Cheng Y.. (2011). Conversion of tryptophan to indole-3-acetic acid by TRYPTOPHAN AMINOTRANSFERASES OF ARABIDOPSIS and YUCCAs in arabidopsis. P. Natl. Acad. Sci. U.S.A. 108, 18518–18523. doi: 10.1073/pnas.1108436108 PMC3215067 22025721

[B94] Wu Y. S. Gong W. Z. Wang Y. M. Yong T. W. Yang F. Liu W. G.. (2018). Leaf area and photosynthesis of newly emerged trifoliolate leaves are regulated by mature leaves in soybean. J. Plant Res. 131, 671–680. doi: 10.1007/s10265-018-1027-8 29600314

[B95] Wu Y. Gong W. Yang W. (2017). Shade inhibits leaf size by controlling cell proliferation and enlargement in soybean. Sci. Rep.-UK 7. doi: 10.1038/s41598-017-10026-5 PMC5569092 28835715

[B96] Wu J. Zhu J. Zhang D. Cheng H. Hao B. Cao A.. (2022). Beneficial effect on the soil microenvironment of trichoderma applied after fumigation for cucumber production. PLos One 17, e0266347. doi: 10.1371/journal.pone.0266347 35917326 PMC9345367

[B97] Xu H. T. Yan L. Zhang M. D. Chang X. L. Zhu D. Wei D. Q.. (2022). Changes in the density and composition of rhizosphere pathogenic fusarium and beneficial trichoderma contributing to reduced root rot of intercropped soybean. PATHOGENS 11. doi: 10.3390/pathogens11040478 PMC9031213 35456153

[B98] Yang F. Huang S. Gao R. Liu W. Yong T. Wang X.. (2014). Growth of soybean seedlings in relay strip intercropping systems in relation to light quantity and red:far-red ratio. Field Crop Res. 155, 245–253. doi: 10.1016/j.fcr.2013.08.011

[B99] Yang F. Liao D. P. Wu X. L. Gao R. C. Fan Y. F. Raza M. A.. (2017). Effect of aboveground and belowground interactions on the intercrop yields in maize-soybean relay intercropping systems. Field Crop Res. 203, 16–23. doi: 10.1016/j.fcr.2016.12.007

[B100] Yasmeen R. Siddiqui Z. S. (2018). Ameliorative effects of trichoderma harzianum on monocot crops under hydroponic saline environment. Acta Physiol. Plant 40. doi: 10.1007/s11738-017-2579-2

[B101] Yuen S. H. Pollard A. G. (1953). Determination of nitrogen in soil and plant materials: Use of boric acid in the micro-kjeldahl method. J. Sci. Food Agr. 4, 490–496. doi: 10.1002/jsfa.2740041006

[B102] Zhai T. Wang Y. Liu C. Liu Z. Zhao M. Chang Y.. (2019). Trichoderma asperellum ACCC30536 inoculation improves soil nutrition and leaf artemisinin production in artemisia annua. Acta Physiol. Plant 41. doi: 10.1007/s11738-019-2836-7

[B103] Zhang H. Duan W. Xie B. Wang B. Hou F. Li A.. (2020). Root yield, antioxidant capacities, and hormone contents in different drought-tolerant sweet potato cultivars treated with ABA under early drought stress. Acta Physiol. Plant 42, 132. doi: 10.1007/s11738-020-03116-x

[B104] Zhang F. Meng X. Yang X. Ran W. Shen Q. (2014). Quantification and role of organic acids in cucumber root exudates in trichoderma harzianum T-E5 colonization. Plant Physiol. Bioch. 83, 250–257. doi: 10.1016/j.plaphy.2014.08.011 25194775

[B105] Zhang F. Yuan J. Yang X. Cui Y. Chen L. Ran W.. (2013). Putative trichoderma harzianum mutant promotes cucumber growth by enhanced production of indole acetic acid and plant colonization. Plant Soil 368, 433–444. doi: 10.1007/s11104-012-1519-6

[B106] Zhao J. (2014). Interplay among nitric oxide and reactive oxygen species. Plant Signaling Behav. 2, 544–547. doi: 10.4161/psb.2.6.4802 PMC2634364 19704554

[B107] Zhao L. Liu Q. Zhang Y. Cui Q. Liang Y. (2017). Effect of acid phosphatase produced by trichoderma asperellum Q1 on growth of arabidopsis under salt stress. J. Integr. Agr. 16, 1341–1346. doi: 10.1016/S2095-3119(16)61490-9

[B108] Zhao L. Zhang Y. (2015). Effects of phosphate solubilization and phytohormone production of trichoderma asperellum Q1 on promoting cucumber growth under salt stress. J. Integr. Agr. 14, 1588–1597. doi: 10.1016/S2095-3119(14)60966-7

[B109] Zhu H. Li X. Zhai W. Liu Y. Gao Q. Liu J.. (2017). Effects of low light on photosynthetic properties, antioxidant enzyme activity, and anthocyanin accumulation in purple pak-choi (Brassica campestris ssp. chinensis makino). PLos One 12, e0179305. doi: 10.1371/journal.pone.0179305 28609452 PMC5469474

